# Rotation with other crops slow down the fungal process in tobacco-growing soil

**DOI:** 10.1038/s41598-024-64735-9

**Published:** 2024-06-19

**Authors:** Ming Liu, Rujun Xue, Chengwei Yang, Ningbo Han, Yanxia Hu, Kaiyuan Gu, Jie Zhao, Shuyue Guan, Jiaen Su, Yonglei Jiang

**Affiliations:** 1 Dali Prefecture Branch of Yunnan Tobacco Company, Dali, 671000 Yunnan China; 2 SouthWest University, Chongqing, 400715 China; 3 Weishan City Branch of Yunnan Tobacco Company, Weishan, 672400 Yunnan China; 4 Yunnan Academy of Tobacco Agricultural Sciences, Kunming, 650021 China

**Keywords:** Tobacco-growing soil, Crop rotation, Fungal diversity, Fungal abundance, Soil fungi process, Ecology, Microbiology

## Abstract

Continuous cultivation of tobacco could cause serious soil health problems, which could cause bacterial soil to change to fungal soil. In order to study the diversity and richness of fungal community in tobacco-growing soil under different crop rotation, three treatments were set up in this study: CK (tobacco continuous cropping); B (barley-tobacco rotation cropping) and R (oilseed rape-tobacco rotation cropping). The results of this study showed that rotation with other crops significantly decreased the soil fungal OTUs, and also decreased the community richness, evenness, diversity and coverage of fungal communities. Among them, B decreased the most. In the analysis of the composition and structure of the fungal community, it was found that the proportion of plant pathogens Nectriaceae decreased from 19.67% in CK to 5.63% in B, which greatly reduced the possibility of soil-borne diseases. In the analysis of the correlation between soil environmental factors and fungal communities, it was found that Filobasidiaceae had a strong correlation with TP and AP, and Erysiphaceae had a strong correlation with TK and AK. NO_3_^−^-N and NH_4_^+^-N were the two environmental factors with the strongest correlation with fungal communities. The results of this study showed that rotation with other crops slowed down the process of soil fungi in tobacco-growing soil and changed the dominant species of soil fungi community. At the same time, crop rotation changed the diversity and richness of soil fungal community by changing the physical and chemical properties of soil.

## Introduction

Tobacco (*Nicotiana tabacum* L.) is an important cash crop widely cultivated worldwide. Its cultivated area has continued increasing in the last few decades. The driving forces behind this trend included the growing demand for tobacco products and the development of associated industries^1^. However, long-term monoculture patterns often lead to a decline in soil quality, biodiversity, and intensification of soil fungal processes^2^. With increasing concerns about environmental sustainability and ecological health, it is critical to explore ways to reduce the reduction in the quality of tobacco-growing soil.

Soil microorganisms played an essential role in various ecological processes of terrestrial ecosystems^3^, including nutrient cycling, pollutant degradation, and balance the stability of ecosystem responses and functions in the era of climate change^4–6^. In the farmland ecosystem, long-term continuous cropping of crops would lead to different soil problems—the long-term constant cropping of cotton increases verticillium wilt disease^7^. Long-term monoculture of yam caused soil acidification and increased the potassium and phosphorus concentrations and soil bacterial richness^8^.

Soil fungal process enhanced the biomass and activity of fungi in rhizospheric soil. The degree of soil fungal process is a vital characteristic to assess the balance and quality of farmland soil^9^. The occurrence and aggravation of various soil-borne diseases in rhizospheric soil with severe soil fungal process^10^. Crop rotation with other crops was considered an effective strategy for crop betterment^11^. By rotating tobacco planting with different crops, root morphology, secretions, and growth conditions could be introduced, thereby changing the ecological environment in the soil and slowing down the fungal process^12–15^.

Although rotation with other crops has been widely acceptable as an advanced agricultural practice for plant diversification, such as barley and oilseed rape were usually chosen as the rotation plan for tobacco because of the suitable growing season and excellent yield. However the slow process of soil fungal in tobacco-growing soil has not yet been fully understood. Limited studies were available on the effects of other crops rotating with tobacco plants on soil fungal communities. Therefore, this study aimed to investigate the impact of different crop and flue-cured tobacco crop rotation on soil fungal process and evaluate the dynamics of soil fungal community under different crop rotation patterns. Compare soil fungal communities with different crop rotation systems to understand the impact of crop rotation with other crops on the fungal process of tobacco-growing soil betterment. The present study also demonstrated an in-depth analysis of the dynamics of fungal communities in soil ecosystems, soil remediation and the promotion of sustainable agricultural development for tobacco-growing areas.

## Materials and methods

### Experiment design

This experiment was conducted at Weishan County, Dali Autonomous Prefecture, Yunnan Province, China (E 100.30, N 25.23, altitude 2000 m) in December 2022. The main physical and chemical properties of the soil were observed as soil bulk density (1.21 g·cm^−3^), pH (6.47), organic matter content (28 g·kg^−1^), total nitrogen content (1.68 g·kg^−1^), total phosphorus (1.46 g·kg^−1^), total potassium (34.54 g·kg^−1^), available phosphorus (18.13 mg·kg^−1^), available potassium (270.23 mg·kg^−1^), and alkaline hydrolyzable nitrogen content (35.23 mg·kg^−1^), respectively.

The field trial employed as randomized block design (RBD) with three biological replicates (*n* = 3). Each plot area covered of 100 m^2^ (10 m × 10 m). All preceding crops in the plots were tobacco cultivar Hongda. The different treatments were as tobacco continuous cropping, cultivar Hongda (CK), barley-tobacco rotation cropping, cultivar Kunlun 15 (B) and oilseed rape-tobacco rotation cropping, cultivar Huayou 5 (R).

The cultivars of barley and oilseed rape are widely used as local varieties. Barley and oilseed rape were sown in December 2022. Row spacing for oilseed rape was 25 cm with plant spacing of 20 cm, and barley row spacing was 25 cm with 10 cm plant spacing. Basal dose of fertilizers were applied before planting, consisting of urea and compound fertilizer. Urea was applied at 10 kg/acre and compound fertilizer (15:15:15) at 12.5 kg/acre. Additional fertilization was carried out in February 2023 with urea (2.5 kg/acre) for barley and oilseed rape crop. No crop cultivation and no fertilizer application was applied in CK treatment condition. All other field management practices followed local field management standards. The crop harvest in May 2023.

### Soil sample collection and analysis

Soil sample collection was carried out after the harvest of crops in May 2023. For each treatment, ten residual crop remnants were selected after harvesting. The roots were excavated, and adhering soil particles on the root surfaces was shaken off. A gentle brushing was used to remove and collect the rhizosphere soil still adhering to the roots. Soil was immediately preserved in liquid nitrogen for the determination of diversity and richness of fungi communities. Another portion was placed in a cool, shaded area for air drying to be used in determination of physical and chemical properties of rhizospheric soil.

Analyzed soil pH, soil organic matter (SOM), total nitrogen (TN), total phosphorus (TP), total potassium (TK), ammonium nitrogen (NH_4_^+^-N), nitrate nitrogen (NO_3_^−^-N), available phosphorus (AP) and available potassium (AK), respectively. Soil NH_4_^+^-N and NO_3_^−^-N were determined by leaching 0.01 mol·L^−1^ CaCl_2_ on automatic flow analyzer (AA3, SEAL, Germany). The TN was decooked with H_2_SO_4_ and accelerators (CuSO_4_ and tin powder) and then determined on a flow analyzer (AA3, SEAL, Germany). TP and AP of the soil were dehydrated with H_2_SO_4_ and HCLO_4_, then observed at 700 nm wavelength on an automatic enzyme label (Infinite 2000, Tecan, Switzerland). The AP was determined on a flame photometer. Taken air-dried soil (5 g) into a triangular bottle, add 50 mL ammonium acetate solution, shaked (30 min), remove and filter for final observation. The content of AK in the soil sample was determined by flame photometer. Soil pH was determined by PH-4 portable pH meter (soil to water ratio, 2.5:1).

Rhizospheric soil samples (*n* = 9) were collected from tobacco-growing soil planted with different precursor crops. Soil fungi DNA was extracted from the soil (0.5 g) by PowerSoil^®^ DNA Isolation Kit (Mobio, AL, USA) according to manufacturer instructions. DNA was measured by NanoDrop 2000 (Thermo Fisher Scientific, Wilmington, DE, USA) and Qubit 3.0 spectrophotometer. The quality of DNA extract was tested using 2% agarose gel electrophoresis^16^. ITS1-1 (5′-CTT GGT CAT TTA GAG GAA GTA A-3′)/ITS1-2 (5′-GCT GCG TTC TTC ATC GAT GC-3′) primer pairs were used to amplify V3–V4 regions of the fungal ITS1 gene, respectively. PCR performed using NEB Phusion High-Fidelity PCR Master Mix following the manufacturer’s recommendations with 30 µg of DNA, 4 μL of PCR primer cocktail and 25 μL of PCR Master Mix. However, negative controls (no templates) were included in this step to check for primer or sample DNA contamination.

PCR products was verified by electrophoresis agarose gel (1%) and purified using AMPure_XP_Beads Kit (AGENCOURT) to remove unspecific products. The library quality was evaluated by an Agilent 2100 bioanalyzer instrument (Agilent DNA 1000 Reagents). The libraries were sequenced using an Illumina HiSeq platform (HiSeq SBS Kit V2, Illumina) platform. Sequence data were deposited in the NCBI SRA database (Accession number: SUB14312496).

### Data analysis

Raw reads with ambiguous bases, an average Phred score less than 20 and length 10 bp were filtered out using Trimmomatic software (v 0.36). The chimeric sequences were identified and removed using UCHIME software (v 4.2.40). The operational taxonomic units (OTUs) of fungi were clustered at 97% sequence similarity using UPARSE (v 7.0.1090). Subsequently, the fungi OTUs were determined by RDP Classifier v 2.2 (Ribosomal Database Project) against the Greengenes (v 201,304) and UNITE (v 7.2) database. Venn plot was shown the number of unique and common OTUs in different groups by the ‘VennDiagram’ package R (v 3.1.1).

The alpha diversity of the fungi communities was characterized by the Chao 1 (species richness) and Shannon (species diversity) indices to analyze the phylogenetic diversity of each group using MOTHUR (v 1.31.2). Moreover, principal coordinate analyses (PCoA) were performed in QIIME software (v 1.80) to reflect the beta diversity of the microbial community, evaluating the similarity in community among the different groups along with the Bray–Curtis distance matrix. Furthermore, linear discriminant analysis (LDA) effect size (LEfSe) was also used to detect significantly different taxa (LDA scores greater than 2.0 at *P* < 0.01) with differential abundance in the Galaxy online analytics platform.

Statistical analysis of the alpha diversity indices of the fungi were performed with Tukey’s honestly significant difference test R package (v 3.5.3) (P < 0.05). The correlation analysis of alpha diversity indices between fungi was used by the function ‘cor. test’ in R package (v 3.5.3) (P < 0.05). One-way analysis of variance (ANOVA) was performed to analyze the impacts of the cultivars on the rhizosphere microbial composition using SPSS. Mantel tests were used to assess the correlation between rhizospheric microbial communities and soil physical and chemical properties (N, P, K, pH and physical properties), as well as temperature using ‘vegan’ package in R (v 3.5.3) (P < 0.05), respectively. In addition, PERMANOVAs was used to assess the effects of fungi community based on the Bray–Curtis distance using the ‘vegan’ package in R (v 3.5.3) (*P* < 0.05). The data were analyzed on the online tool of Majorbio Cloud Platform (https://cloud.majorbio.com/page/tools/).

## Results

### Diversity and richness analysis of fungi community in tobacco-growing soil

The alpha-diversity of soil fungal community under different treatments was shown in Table 1. The results showed that the rotation with other crops significantly reduced the OTUs of soil fungi, and the B treatment was lower (31.21%) than CK. At the same time, compared with the continuous cropping of other crops and tobacco, the community richness, evenness, diversity and coverage of fungal communities in tobacco-planting soil had significant differences. Ace (24.25%), chao (24.35%) and sobs (31.21%) in B treatment were significantly lower than CK, respectively. CK coverage was found higher than B (2.02%) and R (2.04%), respectively. Similar to coverage, Shannon of CK was significantly higher than other treatments (B, 26.62% and R, 13.18%).

**Table 1:** Influence of alpha diversity on tobacco-planting soil fungal community.

Treatment	OTUs	Ace	Chao	Coverage	Shannon	Simpson	Sobs
CK	549.0 ± 74.05^a^	592.57 ± 51.26^a^	584.58 ± 58.06^a^	0.99 ± 0.01^a^	4.02 ± 0.26^a^	0.05 ± 0.01^a^	549.00 ± 60.46^a^
B	377.67 ± 92.91^b^	448.90 ± 80.18^b^	442.24 ± 90.13^b^	0.97 ± 0.01^b^	2.95 ± 0.47^b^	0.11 ± 0.04^a^	377.66 ± 75.86^b^
R	538.33 ± 66.42^a^	624.67 ± 46.82^a^	609.99 ± 46.23^a^	0.97 ± 0.01^b^	3.49 ± 0.39^b^	0.08 ± 0.04^a^	538.33 ± 54.23^a^

**Figure 1:**
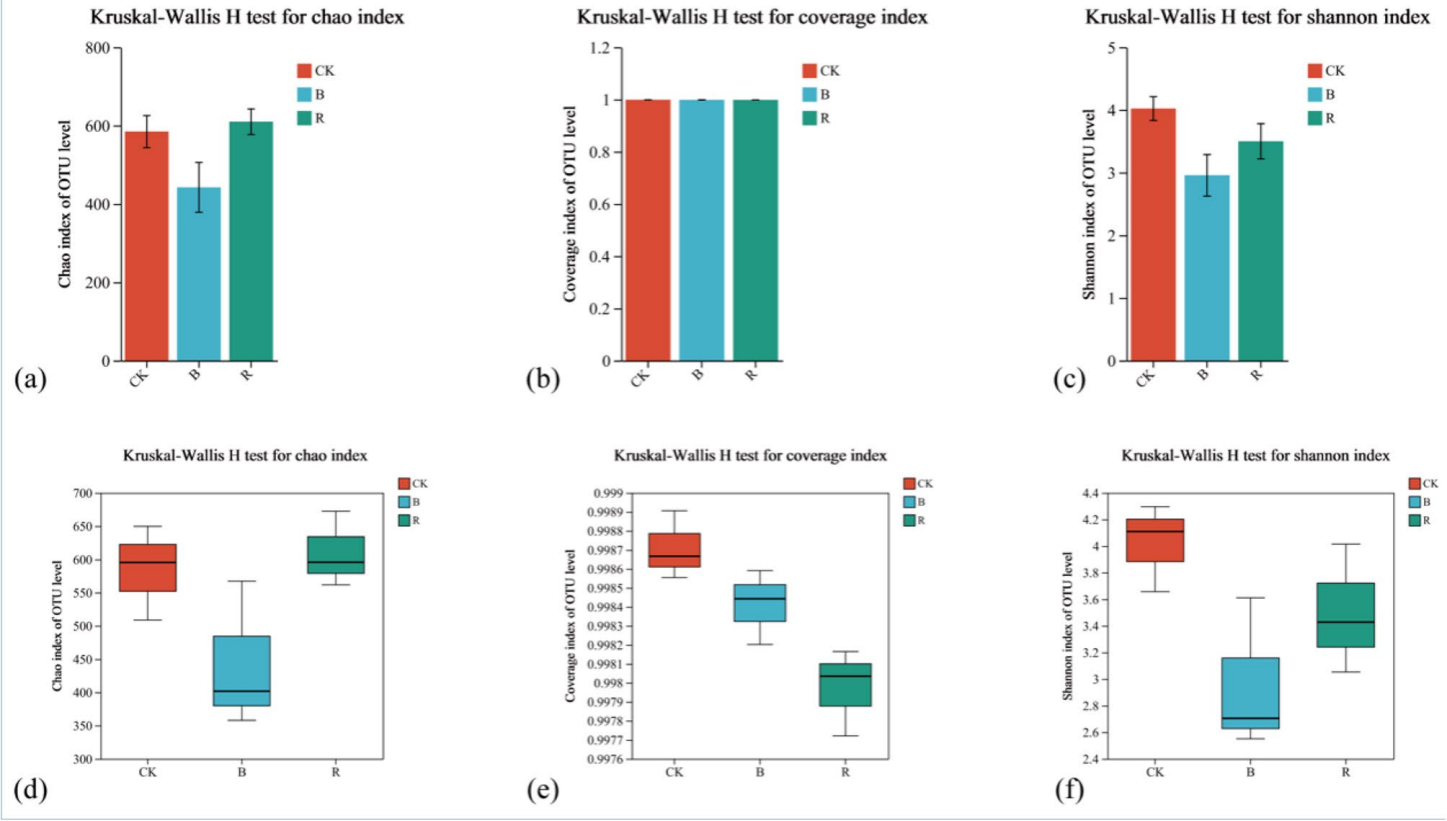
Analysis of fungi alpha-diversity in tobacco-planting soil under different treatments.

**Figure 2:**
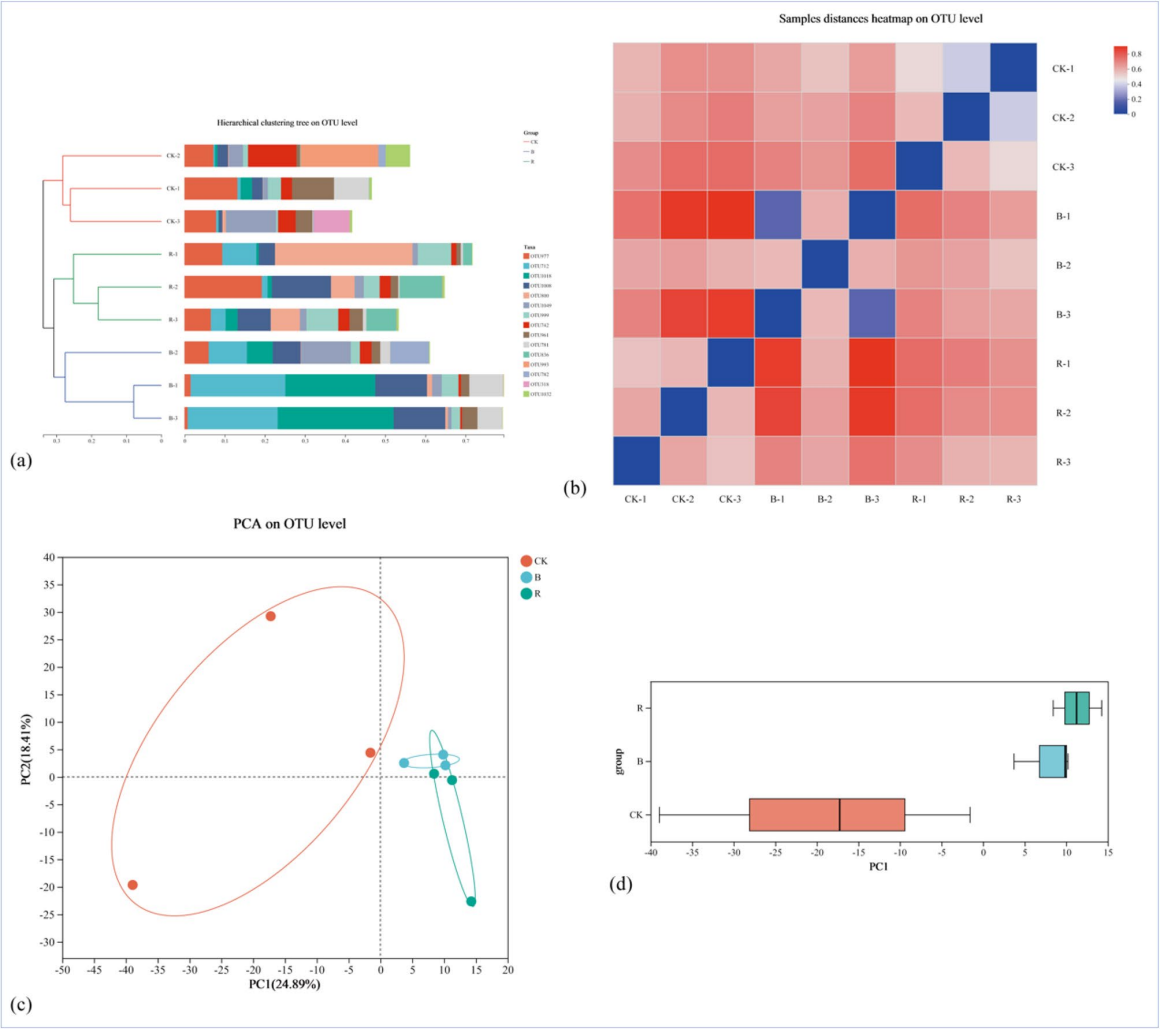
Analysis of fungi Beta-diversity in tobacco-planting soil under different cropping systems.

The column and box chart analysis of chao, coverage and Shannon showed the community richness, evenness, diversity and coverage of the fungal communities in CK higher than B and R tobacco-growing soil (Fig. 1). Clustering analysis was performed on the distance matrix of tobacco-growing soil fungal community (Fig. 2a). The sample level clustering results showed that the samples divided into three significantly different groups according to fungi, indicating that there were significant differences in the composition of fungal community in tobacco-planting soil of different previous crops.

The results indicated that the composition of fungi community in CK, B and R soil was different. The distance heatmap also showed that the composition of fungal communities under the same treatment was similar (Fig. 2b). PCA analysis was performed on the fungal communities of the different treatments, and the similarity and difference of the fungal communities of tobacco-growing soil under different treatments were shown by using two-dimensional visual scatter plots, showing the differential changes of the fungal communities of the tobacco-growing soil (Fig. 2c,d).

### Species composition and difference analysis of fungal community

**Figure 3:**
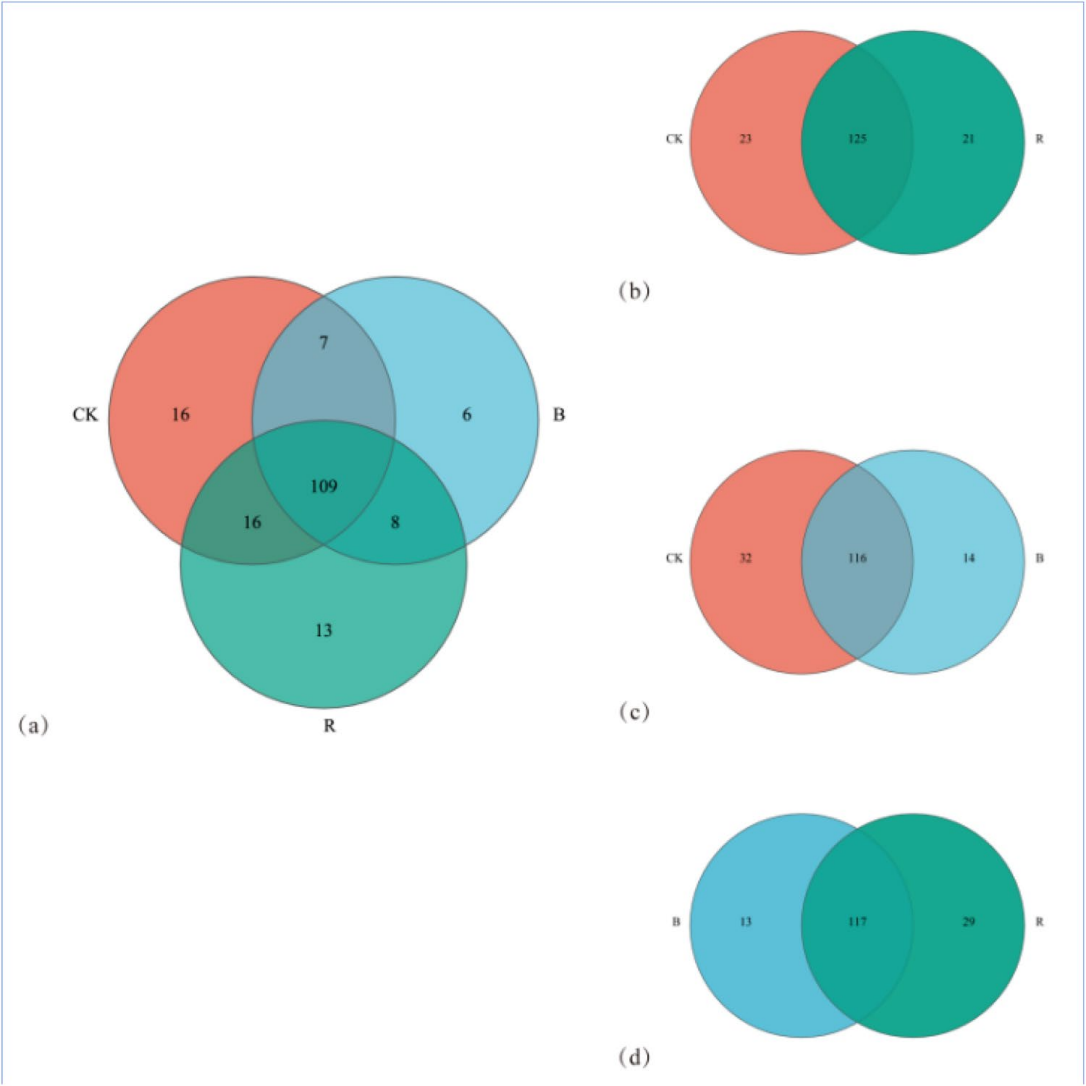
Venn diagram analysis of fungal community composition in tobacco-growing soil under different treatments.

The species composition and differences of tobacco planting soils under different treatments were monitored. The analysis of OTU, 148 families were isolated from CK, 130 families from B, and 146 families from R treatments (Fig. 3). The isolated fungi from B were significantly lower than CK. According to the comparison of the different treatments, the total fungi were divided into 109 families, such as 16 families of CK, 6 families of B, and 13 families of R. The comparison between CK and R showed that 125 families were common, 23 specific to CK, and 21 specific to R. The comparison between CK and B showed that 116 families were common, 32 families specific to CK and 14 families specific to B. The comparison between B and R showed that 117 families were common, 13 specific to B, and 29 specific to R.

**Figure 4:**
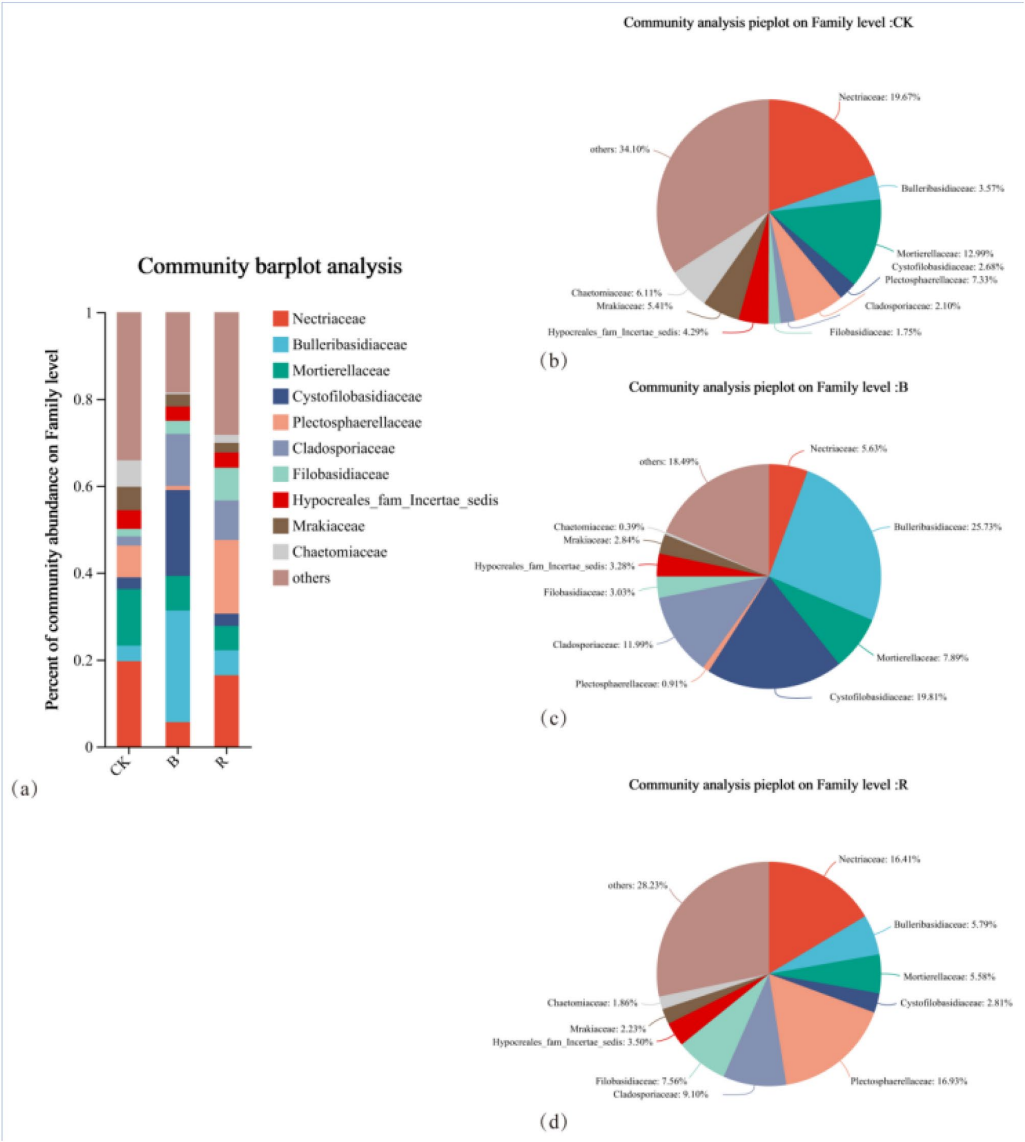
Analysis of fungal community composition in tobacco-growing soil under different treatments.

Figure 4 showed the top ten dominant strains of fungi under different treatments at the family level of tobacco-growing soil and the relative proportion of Top 10 dominant strains in the total sequence under each treatment. There was no significant difference in the dominant strains under different treatments, but the dominant strains with the highest proportion were different. *Nectriaceae* in CK was the most dominant strain in the tobacco-growing soil under this treatment, accounting for 19.67% of the total sequence, and *Mortierellaceae* was the second most dominant strain, accounting for 12.99% of the total sequence.

**Figure 5:**
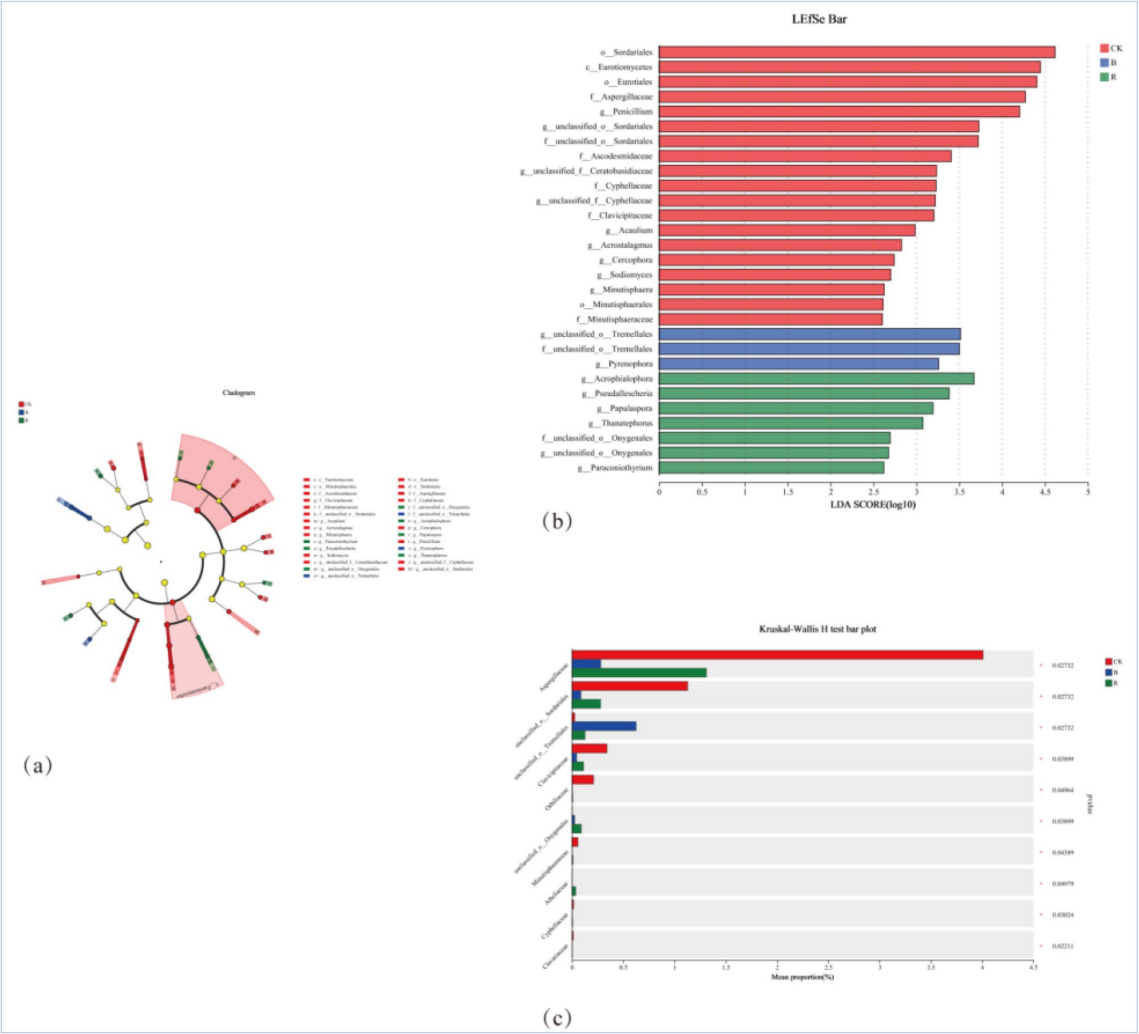
Analysis of fungal community differences in tobacco-growing soil under different treatments.

*Bulleribasidiaceae* is the most dominant strain in tobacco-growing soil during B treatment, accounting for 25.73% of the total sequence, and the second dominant strain was *Cystofilobasidiaceae*, accounting for 19.81% of the total sequence. *Plectosphaerellaceae* was the most dominant strain in R condition, accounting for 16.93% of the total sequence, and *Nectriaceae,* the second dominant strain, accounting for 16.41% of the total sequence. The species differences at different taxonomic levels in the form of a developmental tree, directly reflected the different species at different species hierarchy levels between different groups (Fig. 5a). The LDA values of different differential species influence of the signature species identified between different groups on the differential effect was directly demonstrated through LEfSe analysis (Fig. 5b). It showed that there were more fungal groups with significant effects in CK, and the abundance of *Sordariales* had the greatest influence on the differential effect.

**Figure 6:**
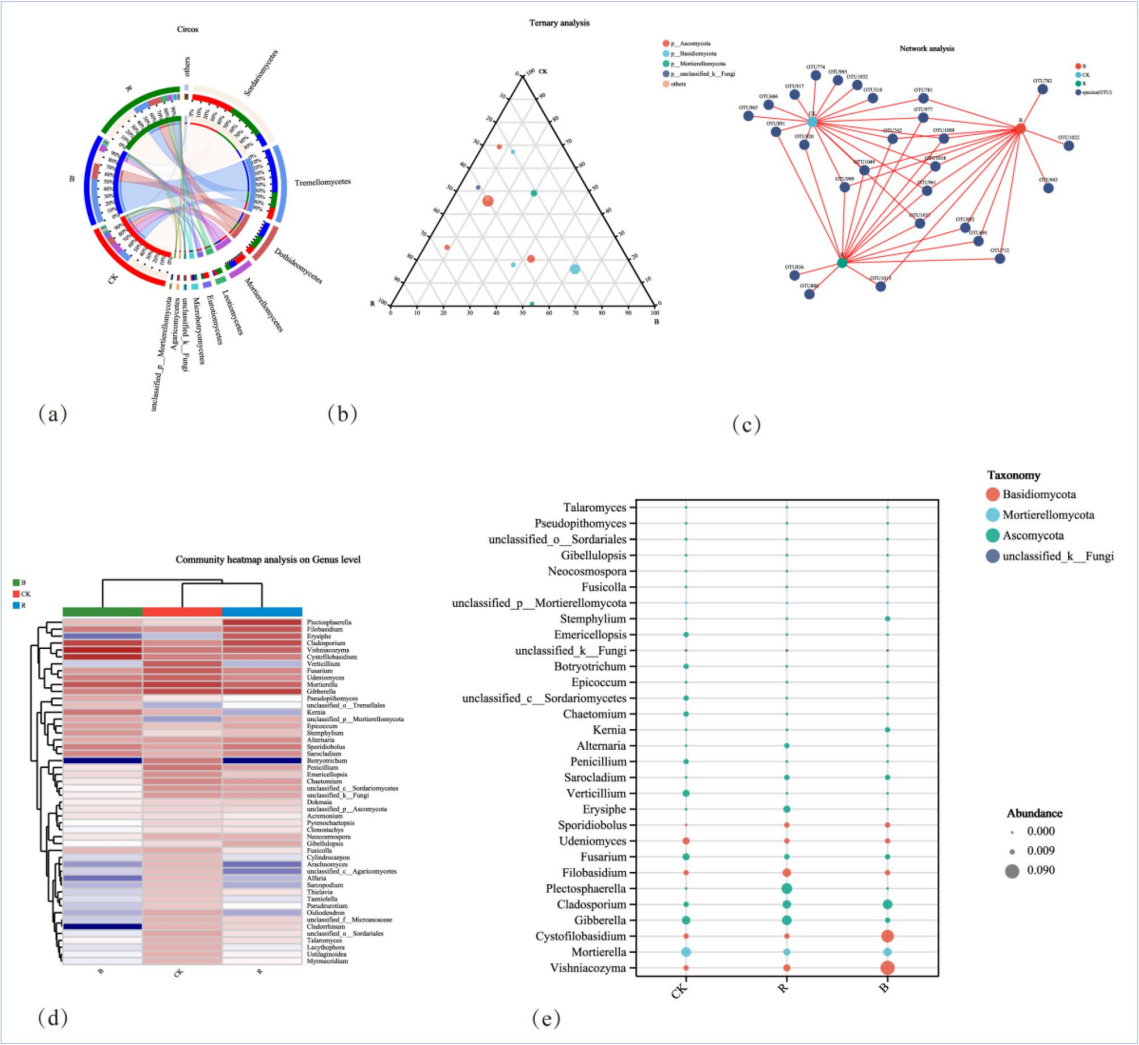
Relationship between species of soil fungi in different previous crops and tobacco-growing soil fungi.

Figure 5c showed the different fungi under different treatments (family level). *Aspergillaceae, Sordariales, Tremellales, Clavicipitaceae* and *Orbiliaceae* were the top 5 with most significant differences. Meanwhile, *Aspergillaceae, Sordariales, Clavicipitaceae* and *Orbiliaceae* in CK were significantly higher than the other treatments. Figure 6 showed the Circos, Ternary, co-occurrence network map, heatmap, and bubble map of the species composition of the tobacco-growing soil fungal community under different treatments. Circos and Ternary analyzed the fungal community at class level, and heatmap and bubble map observed the fungal community at genus level. The results of Network analysis showed that the 25.92% of OTUs were related to CK and 11.11% of OTUs related to B only. The OTUs related to R only (7.41%), and the OTUs related to CK (66.67%), which was significantly higher than B and R. The distribution analysis of the top dominant species under different treatments at class level, it was found that *Sordariomycetes* and *Tremellomycetes* accounted for the larger two species, and *Sordariomycetes* accounted for 24% of all classes in CK (Fig. 6a,b). 33% of B and 43% of all classes in R. *Tremellomycetes* represented 54% of all classes in CK, 27% of in B, and 20% of in R treatment.

The Ternary phase diagram revealed the fungi composition characteristics of 9 classes of tobacco-growing soil samples under the different treatments. Circles of the same color in the figure represented the same class, and the size of the circle area represented the size of the abundance. The results showed that the composition and distribution ratio of microorganisms in different samples were different, and the dominant strains under each treatment also different. When analyzing the distribution of top dominant strains under different treatments at genus level (Fig. 6c–e). It was found that CK and R were similar in the composition of top dominant strains, while CK and B were significantly different.

### Correlation analysis of soil fungal community and soil environmental factors

**Figure 7:**
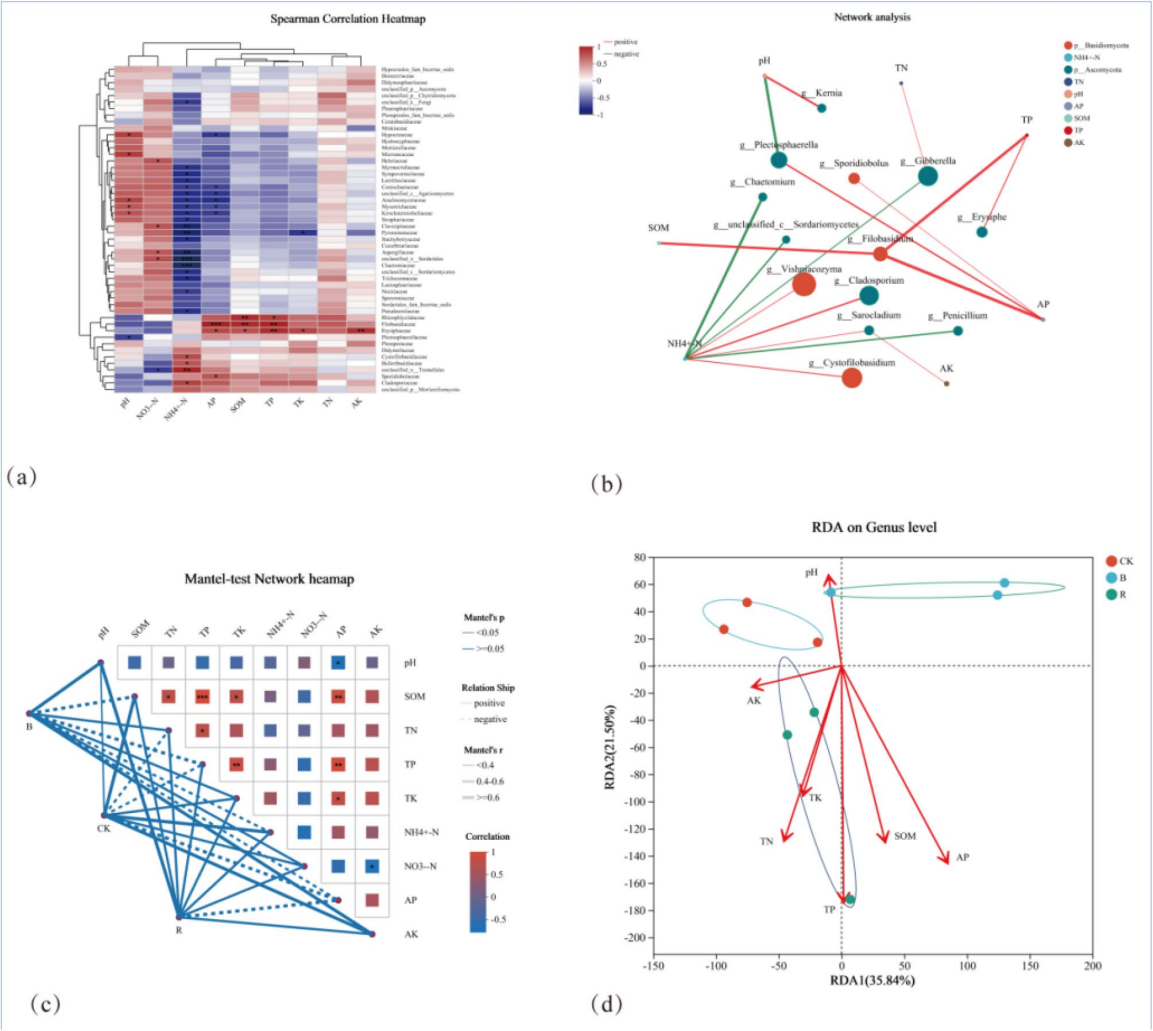
Correlation analysis of tobacco-planting soil and soil environmental factors.

Correlation analysis was conducted on the fungal community of tobacco-planting soil and soil environmental factors (Fig. 7). The heatmap showed the correlation coefficient between the environmental factors of tobacco-planting soil and the top 50 dominant fungi. *Filobasidiaceae* was strongly correlated with TP and AP, and *Erysiphaceae* was strongly correlated with TK and AK. As for environmental factors, NO_3_^−^-N and NH_4_^+^-N were found the strongest correlation with fungal communities, and strong correlation with *Sordariales, Clavicipitaceae, Tremellales, Aspergillaceae*, etc. Network analysis showed correlations between fungi and environmental factors in *Basidiomycota* and *Ascomycota* (only *P* < 0.05 species).

*Filobasidium* was strongly positively correlated with SOM, TP and AP, while NH_4_^+^-N was correlated with *Basidiomycota* and *Ascomycota*. Mantel-test network heatmap analyzed the correlation between environmental factors and the correlation between fungal communities and environmental factors under different treatments. The lines in the figure represented the correlation between community and environmental factors, and the heatmap represented the correlation between environmental factors. The results showed that the fungal community under CK treatment was positively correlated with pH, SOM, TK, AK, NH_4_^+^-N and NO_3_^−^-N, and negatively correlated with TN, TP and AP.

Under B condition, the fungal community was positively correlated with pH, TN, TK, NO_3_^−^-N and AK, and negatively correlated with SOM, TP, NH_4_^+^-N and AP. The fungal community was positively correlated with pH, SOM, TK, AK, NH_4_^+^-N, NO_3_^−^-N, TN, TP, and only negatively correlated with AP during R condition. Only CK was negatively correlated with TN, while B and R positively correlated with TN.

## Discussion

### Rotation with other crops downregulate the enhancement in the diversity and richness of fungal communities in tobacco-growing soil

Fungi play a potential role in the soil ecosystems. It involved in soil nutrient circulation, organic matter decomposition, and plant pathology, and they are closely associated to the plant growth, development and production^17^. Certain fungus in the soil were plant pathogens that cause plant pathogenic diseases, whereas some others were biocontrol factors that can gain or loss the impact of plant diseases^18–20^. Soil fungal community structure is obvious impacts by the soil physico-chemical profile. Soil pH and enzyme activity are strongly linked with rhizospheric fungal communities as well. In agroecosystems, agricultural management strategies significantly influenced the soil fungal communities^17,20,21^.

The different soil fungal diversity and community structure characteristics were formed the combined effects of planting system and previous crop types. Alpha-diversity analysis was found optimum community richness, evenness, diversity and coverage of the fungi community in tobacco soil under continuous cropping, which was correlated to the farming system. Soil fungal richness of soybean increased with the continuous cropping years^22^. Continuous cropping resulted in the loss of soil bacteria to fungi ratio^23^. After continuous cropping, autotoxic substances accumulated continuously in rhizosphere soil, the structure of fungal community changed, and harmful fungi such as *Alternaria* and *Fusarium* were enriched^24^.

Different planting patterns affected the type, quantity and quality of plant residues. Plant residues are the source of nutrients for microorganisms, and different plant residues could lead to changes in the function of soil microorganisms^25^. The result findings showed that the diversity and richness of fungal community in tobacco-growing soil were significantly different under rotation and continuous cropping, and rotation with other crops slow down the increase of fungal community diversity and richness in tobacco-growing soil. The selection of crop rotation also affected the diversity and richness of fungal community in tobacco-growing soil. The chao, Shannon, richness and diversity of rape growers were high, and the community coverage of barley also high.

The different crop rotation patterns had different effects on soil physicochemical properties and microbial diversity, and could gain or loss the abundance of harmful or beneficial microorganisms^26^. Previous studies confirmed the results of this study as the selection of different crop rotations affected the fungal community in tobacco-growing soil in different ways.

### Species composition and difference analysis of tobacco-growing soil under different crop rotation

The rotation with other crops changed the dominant strains in tobacco-growing soil. Among the fungi in tobacco-growing soil under continuous cropping, *Nectriaceae* was the most dominant species, and *Nectriaceae* generally plant pathogens, which could cause the canker disease of apple and other fruit trees. The long-term continuous cropping of tomato would lead to increase the relative abundance of *Gemmatimonas* and intensification of the degree of disease^27^.

The long-term continuous cropping of tobacco would lead to the occurrence of diseases^28^. The results of this study showed that the pathogenic fungi became the most dominant species in the soil fungal community under continuous cultivation of tobacco, which explained the cause of serious disease in the tobacco field from the perspective of soil microbiology. LEfSe map showed that the accumulation of fungal species in tobacco-growing soil under continuous cropping was significantly more than rotation cropping. The soil-borne diseases were mostly caused by fungi, and the fungal community composition of soil with different disease occurrence^29,30^.

The fungal community structure of tobacco-growing soil was modified by the crop rotation, and pathogen community in tobacco-growing soil was no longer dominant, which slow down the process of soil fungal progress. Legume crop rotation patterns significantly changed fungal diversity and community composition^31^. It showed that the proportion of *Aspergillaceae, Sordariales, Clavicipitaceae* and *Orbiliaceae* in tobacco-growing soil significantly reduced under rotation with other crops.

The species of highly diverse fungal genus *Aspergillus* were well-known agricultural pests and producers of various mycotoxins threatening food safety worldwide^32^. The rotation with other crops can change the composition of fungal species in tobacco-growing soil and reduced the number of pathogenic bacteria, thereby maintaining soil health and reducing crop morbidity.

### Correlation analysis of soil fungal community and soil environmental factors

Tobacco-growing soil environment directly affect the growth and development of tobacco, and also the major external factor affecting soil microorganisms. The analytical relationship between fungi community in tobacco-planting soil and soil environmental factors was helpful to understand how rotation with other crops slow down the fungal process of tobacco-growing soil by changing the physical and chemical properties of the rhizospheric soil. The physical and chemical properties of tobacco-growing soil may be changed by crop rotation with different crops, thus affecting the fungal community in tobacco-growing soil. The bulk density, organic matter, total nitrogen, total phosphorus and total potassium contents of tobacco-growing soil were significantly different under the two different planting modes of mushroom + tobacco and rice + tobacco^33,34^.

Compared with tobacco continuous cropping, rotation with other crops increased soil TN content. Planting of different crops had different effects on the nutrient content of tobacco-growing soil. It showed that the NO_3_^−^-N and NH_4_^+^-N were the environmental factors strongly correlated with fungal communities. The environmental factors affecting the expression levels of soil enzymes and genes related to nitrogen cycle^35^. These results indicated that the most important factors affecting soil microenvironment among soil environmental factors. Different crop rotations affected soil microenvironment by changing soil nutrient profile and reduce the soil fungal process.

## Conclusion

This study showed that crop rotation with other crops could slow down the soil fungal process in tobacco-growing soil by changing the fungal richness and community composition. The crop rotation with other crops reduced the richness of the fungal community in tobacco-planting soil, and the composition of fungi in tobacco-growing soil changed so that pathogenic fungi were no longer the dominant species in tobacco-growing soil fungi. Long-term continuous cropping fostered synergistic enhancements in potential plant pathogenic fungi and significant microorganisms. However, further research is warranted to assess whether and how pathogenic and beneficial microorganisms interact with plant secondary metabolites.

### Ethical consideration

The tobacco seed collection and the trial conducted in this study were in no violation of any legislation, including the IUCN Policy Statement on Research Involving Species at Risk of Extinction and the Convention on the Trade in Endangered Species of Wild Fauna and Flora. The tobacco seeds in this research were provided by Professor Jiaen Su, who was employed by Dali Prefecture Branch of Yunnan Tobacco Company. The collection was permitted by the local government of the aforementioned sites.

## Data Availability

The original contributions presented in the study are included in the article, further inquiries can be directed to the corresponding author/s. Sequence data were deposited in the NCBI SRA database under accession number SUB14312496. And the website was: https://submit.ncbi.nlm.nih.gov/subs/sra/SUB14312496/overview.
